# The Effect of Life Skills Training on Mental Health of Iranian Middle School Students: A Preliminary Study

**Published:** 2016-10

**Authors:** Saman Jamali, Sekineh Sabokdast, Hamid Sharif Nia, Amir Hossein Goudarzian, Sima Beik, Kelly-Ann Allen

**Affiliations:** 1 Student Research Committee, Sari Faculty of Nursing, Mazandaran University of Medical Sciences, Sari, Iran.; 2 Islamic Azad University of Sari, Sari, Iran.; 3 Assistant Professor, Department of Medical-Surgical Nursing, Amol Faculty of Nursing, Mazandaran University of Medical Sciences, Sari, Iran.; 4 Biostatistics Student, Mazandaran University of Medical Sciences, Sari, Iran.; 5 The Melbourne Graduate School of Education, The University of Melbourne, Melbourne, Australia.

**Keywords:** *Iran*, *Life Skills*, *Mental Health*, *Students*, *Training*

## Abstract

**Objective:** This research aimed to study the effect of life skill training on mental health of Iranian middle school students.

**Method:** This experimental study was conducted In 2015 on 100 students of city of Ghaemshahr (North of Iran), who were randomly selected and divided into two equal groups of intervention (n = 50) and control (n = 50). Qualified trainers provided eight sessions (two sessions a week for 2 hours) of life skills training to the intervention group for one month. The control group did not participate in any training sessions during the same period. Mental health in both groups was assessed by a questionnaire pre- and post-training. Data were analyzed using descriptive and infernal (ANCOVA and paired t-test) statistic tests.

**Results:** The average age of the participants in both groups was 13.5±1.01. ANCOVA test results revealed that the average score of violence, addiction, stress and sensation-seeking before and after the training was statistically significant in the intervention group and control groups (p<0.001).

**Conclusion**: Life skills training had positive effects on mental health of the participants. Given the importance of mental health in modern societies, it is necessary for schools to incorporate life skills in their curriculum to support the mental health of adolescents.

According to the World Health Organization (WHO), mental health is defined as “a state of well-being in which every individual realizes his or her own potential, can cope with the normal stresses of life, can work productively and fruitfully, and is able to make contribution to his or her community (1). WHO in the "Health 2020" program states that mental health improvement involves making people flexible against various stressors in life (2-4). Flexibility is defined as the capacity for a person, group or community to avoid, overcome or minimize the damaging effects of stress from daily life experiences (e.g., facing an unfamiliar situation) (4, 5). Research shows that mental health promotion is more effective when a person is taught skills of coping, adaptability, and flexibility early in life (6, 7). Evidence suggests that programs concerned 

with social-emotional learning that address stress management, coping and life skills that are implemented in elementary, middle and high schools can have a positive impact on mental health and reduce risk factors for emotional and behavioral problems later in life (8, 9(.WHO has provided a set of guidelines for life skills that are concerned with the mental, social and interpersonal skills of individuals (10). Life skills empower individuals to have more appropriate and flexible behavior in the community and increase their self-esteem (11). Some studies have found positive effects of life skills training on responding to psychosocial and social factors such as depression, anxiety, low self-esteem, anger, addiction and interpersonal conflict (12-14).

Although the positive effect of life skills on students’ mental health has been noted in several studies, it did not show good effects in some researches, (12, 14-16). Thus, due to these conflicts, more studies are needed to assess the relationship between these variables brightly. Furthermore, given the importance of life skills training for young people, this type of intervention should be provided for all students (17). Moreover, based on available databases, no study of similar nature has been conducted on students of Mazandaran province. In addition, this was the first survey that contains comprehensive dimensions of mental health against other studies (12, 18 and 19). Thus, the purpose of this study was to determine the effect of life skills training on the mental health of middle school students in Mazandaran province (Northern Iran).

## Materials and Method

***Procedure***

A random sample of 100 middle school students in the city of Ghaemshahr was used in the current study (14). The participants were randomly divided into two 50-member groups of intervention and control. The study was approved by the Ethics Committee of Mazandaran University of Medical Sciences (MUMS) (Code 252). Inclusion criteria included willingness to complete a full mental health assessment (assessed by a psychologist) and an age range between 13 and 14 years. Participation began after obtaining written consent from parents and students. 

***Intervention Protocol***

The intervention group received life skills training for one month in eight sessions (two sessions a week for two hours) by qualified teachers (12, 13). The followings skills were taught in two sessions: Empathy, problem solving, critical thinking, coping skills, self-regulation and assertion skills. The training method involved lecture-style presentations, group activities, role-play and question and answer opportunities. 

***Materials***

Mental health in both groups was assessed by a questionnaire before and after training. This tool consisted of four categories including stress (based on Kettle Personality Scale), sensation-seeking (based on Zuckerman scale), violence (based on Buss and Perry Aggression Questionnaire (BPAQ)] and attitude to tobacco addiction), which resulted in a total of 40 questions. Attitude to tobacco addiction scale was adapted based on the standard scale of Rezaei, et al. (2012) study (20). Qualitative content validity of the questionnaires was assessed by medical sciences experts in Mazandaran University (five nursing doctorates, two psychiatrists and two clinical psychologists) (21). Items were revised the items according to their comments; its reliability was assessed by test-retest method on 30 students at an interval of two weeks and was confirmed at 0.80. Also, the Cronbach's alpha for each category of the questionnaire (violence [α = 0.8], attitude to tobacco addiction [α = 0.78], stress [α = 0.76] and sensation-seeking [α = 0.77]) was calculated.

***Statistical Analysis***

An Analysis of covariance (ANCOVA) was conducted to examine the effect of life skills training on the mental health of the participants (SPSS, Version 23.0). Also, paired t-test was used to examine the score changes before and after the intervention and their effect sizes in both groups. The significance level in all the tests was set at p < 0.05.

## Results

The study was conducted using the responses of 100 middle school students. The average age of the students in both intervention and control groups was 13.5±1.01; and half of the students (50%) were male. There were no differences between the groups (P>0.05). ANCOVA test results (Table 1) showed that the average score of violence after training in the intervention (18.24) and control groups (22.08) was statistically different, F (2, 97) = 9.56, (p) <0.001, η2 = 0.165.Moreover, the mean scores of the addiction factor in the intervention group (17.90) and control group (21.96) was statistically different, F (2, 97) = 9.93, (p) <0.001, η2 = 0.170.

**Table1:** Score Changes of Variables after Life Skills Training in Students

**Variables**	**Intervention**		**Control**		**F**	**MS**	**P**
	**Mean**	**SD**	**Mean**	**SD**			
Violence	18.24	2.44	22.08	6.37	10.401	234.109	<0.001
Stress	18.48	2.48	22.18	6.35	14.699	324.25	<0.001
Addiction	17.90	3.24	21.96	5.56	19.870	412.09	<0.001
Sensation-Seeking	25.20	2.92	29.02	5.47	18.906	364.81	<0.001
Total	79.82	7.36	95.24	19.74	26.764	5944.41	<0.001

**Table2:** Mean Differences Score of Mental Health in Control and Experimental Group Before and after the Intervention

**Variables**	**Pre-test**	**Post-test**	**t**	**P**	**Effect Size**
	**Mean (SD)**	**Mean (SD)**			
Violence	26.09 (3.35)	20.16 (5.17)	10.261	<0.001	1.026
Addiction	24.89 (7.26)	19.93 (4.96)	5.783	<0.001	0.578
Stress	26.83 (3.73)	20.33 (5.14)	10.365	<0.001	1.036
Sensation-Seeking	31.89 (7.26)	27.11 (4.77)	5.660	<0.001	0.56
Mental health	109.70 (16.38)	87.53 (16.73)	10.318	<0.001	1.032

The mean scores of the stress factor in the intervention group (18.48) and control group (22.18) was statistically different, F (2, 97) = 6.15, (p) <0.001, η2 = 0.113. The mean scores of sensation-seeking factor in the intervention group (25.20) and control group (29.02) was statistically different, F (2, 97) = 9.52, (p) <0.001, η2 = 0.164. The mean of total scores of the questionnaire in the intervention (79.82) and control groups (95.24) was also statistically different, F (2, 97) = 16.14, (p) <0.001, η2 = 0.250. The mean differences score of mental health in both groups before and after the intervention is demonstrated in Table 2. Most variables had >1 effect size level.

## Discussion

The results of this study revealed that in general, life skills education for middle school students had a significant effect on four variables of mental health (stress, violence, addiction, sensation-seeking) (Eta-2 = 0.245; p<0.001). In other words, life skills are effective in reducing drug addiction, violence and stress and sensation-seeking. We reviewed many studies conducted on the effect of life skills training in various aspects of mental health (19, 22 and 23). In one research, it has been found that life skills training had significant effects on depression, anxiety and stress among students, which is consistent with the results of our study as they relate to the factor of stress (24, 25). In a longitudinal study of university students in Sweden, it was shown that stressors had a negative effect on students' mental health and they were significant barriers for academic achievements of the students (26).

In another study, it was found that life skills training for patients with non-metastatic breast cancer was an effective method in reducing symptoms of depression, anxiety, sleep disorders and physical and social dysfunction (13).

In general, training of problem solving skills has been effective in increasing social competence of students in behavioral, emotional and motivational subscales, and teaching students such skills at schools can be effective in improving their social competence (27).

Previous studies have also shown that training in life skills in different situations, training programs, age groups and circumstances could reduce the use and abuse of alcohol and drugs in the long term (10, 23, 28 and 29) and that life skills training can be effective in reducing health risk behaviors, increasing academic achievement and material savings (23). In a study by Luna-Adame and colleagues, 28 Spanish students (14 students per group) were examined and the experimental group received 21 hours of training in the first semester and 12 hours in the second semester. According to the results, life skills training did not have a significant effect on reducing the dependency of the drug, which was inconsistent with the present findings (12). Possible reasons for this difference may include differences in cross-cultural conditions of the communities, the environment of research, teaching methods and small sample size. Offering effective programs to prevent drug abuse help students to improve their knowledge and social confidence, and empower them to resist the use of alcohol, tobacco and other substances (30). Other researches with the aim of determining future prospects of life skills-based education have shown that the etiology of aggression and substance use in adolescents was almost similar and this behavior usually occurs at the same time in adolescents. It also stated that the teaching of life skills has a significant effect on reducing the amount of physical and verbal aggression, conflict and crime (31). Providing life skills training program for middle schoolers can provide new opportunities for them to improve their critical thinking and problem solving skills, cope with emotions and stresses and improve their ability to say no (29). However, the results of programs based on the life skills training approach have been controversial, with some authors finding no preventive effects on addiction (15, 32 and 33). These inconsistent results may be due to the differences in the education procedure and study samples.

## Conclusion

According to the results, life skills training had a considerable effect on mental health parameters. Considering the significance of mental health in modern societies and particularly in adolescents, it is of importance to incorporate these skills in school curriculums and hold workshops for parents to improve the mental health of the adolescents.
